# Social Support, Attachment Closeness, and Self-Esteem Affect Depression in International Students in China

**DOI:** 10.3389/fpsyg.2021.618105

**Published:** 2021-03-04

**Authors:** Yawen Li, Fei Liang, Qiuyue Xu, Simeng Gu, Yansong Wang, Yang Li, Zhi Zeng

**Affiliations:** ^1^School of Health Economics and Management, Nanjing University of Chinese Medicine, Nanjing, China; ^2^Institute of Brain and Psychological Science, Sichuan Normal University, Chengdu, China; ^3^School of Medicine, Nanjing University of Chinese Medicine, Nanjing, China; ^4^Department of Psychology, Jiangsu University Medical School, Zhenjiang, China; ^5^Department of Psychology, Nanjing Forest Police College, Nanjing, China

**Keywords:** social support, depression, attachment and closeness, self-esteem, international students in China

## Abstract

With an increase in the number of international students in China, there has been a simultaneous increase in their emotional problems, such as depression, as well as the importance of their emotional well-being. This study aimed to investigate the influence of social support on depression and the mediation and moderation mechanisms of this relationship in international students. In total, 349 international students in China responded to a questionnaire survey comprising the Social Support Rating Scale, Self-rating Depression Scale, Adult Attachment Scale, and Self-Esteem Scale. The results showed that: (1) attachment closeness had a significant direct predictive effect on depression; (2) attachment closeness played a mediating role in the relationship between social support and depression; and (3) the direct effect of social support on depression and the mediating effect of attachment and closeness are regulated by self-esteem. Therefore, interventions aimed at improving the social support, attachment closeness, and self-esteem of international students in China can be effective in reducing their depressive symptoms.

## Introduction

As the influence of China’s international education continues to increase, the number of international students coming to China has also increased significantly. With the upsurge in the number of international students, the problem of depression among this group has become increasingly prominent. At the same time, international students in China need to adapt to the Chinese environment. Specifically, such adaptation is even more important during the current times as we face the coronavirus disease 2019 (COVID-19) pandemic. During the COVID-19 pandemic, international students can only avoid depression if they adapt well to the Chinese environment and culture. Even under regular circumstances, international students are more prone to mental disorders (e.g., depression) and less motivated to seek psychological service than their domestic peers (Alharbi and Smith, 2018; Brunsting et al., 2018). In this scenario, finding ways to effectively control depressive symptoms in international students is related not only to students’ interests and well-being but also to the international image of China’s higher education system and social stability. Therefore, we deemed that examining the unique mechanism that affects depressive symptoms in international students in China can be of considerable importance, since such knowledge can allow stakeholders to propose, develop, and apply effective intervention strategies to deal with this issue.

Depression is a psychological disorder, with several implications for physical health as well, that has been seriously damaging human health in modern society (Chong et al., 2020). Its main clinical symptoms include marked and persistent depressive emotions, which are usually caused by the sudden occurrence of major life-changing events or long-term nervous and unpleasant emotional experiences. Previous studies have found that depressive symptoms are a key issue affecting cross-cultural adaptation in international students (Smith and Khawaja, 2011; Schofield et al., 2016), and compared with local students, international students showed more severe depression (Liu et al., 2016). Accordingly, these severe symptoms may not only affect the social and academic activities of international students (Kernan et al., 2008; Orth et al., 2014) but also lead to suicidal thoughts and behaviors if they develop to a serious stage (Ross, 2010).

Depression is closely related to the social environment (Rutter, 2005; Lev-Ran et al., 2014), and social support is an important environmental factor. Previous studies have shown that positive social support cannot only enhance self-awareness and reduce psychological stress responses (Sarason et al., 1991) but also buffer the negative effects of stressful events (Chiang et al., 2018; Lau et al., 2018). Some studies have also found that social support levels are significantly related to depression severity. A high level of social support can hinder the occurrence of depression (Shen et al., 2019; Scott et al., 2020); contrarily, low social support may lead to continuous and severe depressive symptoms (Morris et al., 1991). Therefore, in the real world, social support can reduce depression (Tham et al., 2020). From a theoretical point of view, social support cannot only provide direct help but also provide emotional support for international students, thereby facilitating the increase of positive emotional experiences and indirectly protecting their physical and mental health. However, access to social support for international students is different than that for native students, mostly because they tend to not have easy access to family members or close relatives in a foreign land. To examine this process, we explored the influence of social support on depression and the underlying mechanisms of this relationship in international students residing in Jiangsu, China.

Attachment is defined as a secure emotional bond between people over time and space (Bowlby, 1969; Ainsworth, 1979). The importance of attachment in adulthood has also been recognized; adults too turn to their attachment figures in times of stress (Robles and Kane, 2014). Attachment closeness refers to people’s perceived comfort when sharing intimacy with others (Collins and Read, 1990). It can be considered that attachment closeness may influence people’s future social support levels. Exemplifying this empirically, studies have shown that individuals with low social support experience lower relationship satisfaction, more interpersonal conflicts, and have a higher risk of depression (Lakey and Orehek, 2011; Hames et al., 2013). Moreover, many studies have confirmed the relationship between depression and low attachment (Aderka et al., 2009; Morley and Moran, 2011). Accordingly, we assumed that attachment closeness plays a mediating role in the relationship between social support and depression in international students in our sample.

Self-esteem refers to experiences of self-respect and self-love that are generated by individuals based on self-evaluation, and it requires perceived respect from others, collective support, and societal approval to function. Additionally, self-esteem is an important psychological component of self-regulation (Mruk, 2006). People’s self-esteem reflects their perceived self-worth and belief in their abilities; for example, a study showed that high self-esteem played an important role in improving psychological adaptability, protecting established relationships, and promoting mental health, whereas low self-esteem was closely related to various issues in interpersonal relationships, adaptation, and psychosomatic problems (Korrelboom et al., 2012). Given the protective effect of self-esteem on mental and physical development, it can be assumed that a low level of social support may influence depression to a diminished extent in individuals with high self-esteem. Therefore, we hypothesized that the mediating effect of attachment closeness is moderated by self-esteem; specifically, compared with individuals with low self-esteem, those with high self-esteem will present a weaker mediating effect.

**FIGURE 1:**
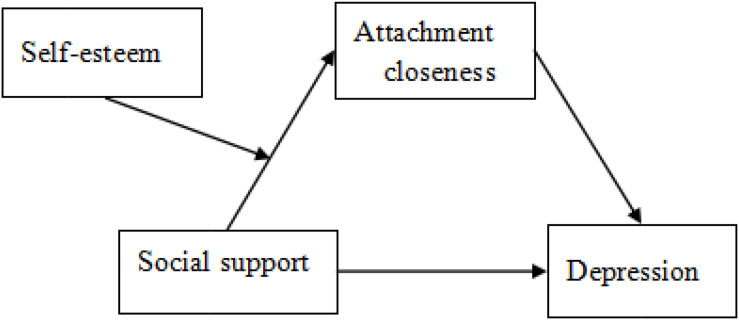
Hypothetical model of the moderated mediation of self-esteem and attachment closeness for the relationship between social support and depression.

To summarize, this study aimed to investigate the influence of social support on depression and the mediation and moderation mechanisms of this relationship for international students in China. Despite the bulk of literature on the relationship between depression, social support, attachment closeness, and self-esteem, most studies have only focused on the relationship between two of these variables; few studies have analyzed the relationship between multiple variables, especially regarding the underlying mechanisms of their relationships. We hypothesized that: (1) attachment closeness plays a mediating role in the influence of social support on depression; (2) the mediating effect of attachment closeness is regulated by self-esteem; and (3) the mediating effect is stronger in high self-esteem than in low self-esteem conditions. The hypothetical model is shown in Figure 1.

## Materials and Methods

### Participants and Procedure

We used a paper-based questionnaire and a convenient sampling method to select international students from Nanjing University of Chinese Medicine and Jiangsu University. The questionnaires were distributed with the assistance of teachers in each institution and collected on the spot. We collected 396 questionnaires, among which 44 were invalid, resulting in a final sample of 349 valid questionnaires. The students’ average age was 20.65 years (*SD* = 2.40); there were 183 (52.4%) men and 166 (47.6%) women. The study was approved by the ethics committee of Nanjing University of Chinese Medicine.

### Measures

#### Self-Rating Depression Scale

We used the 20-item English version of the Self-rating Depression Scale (SDS), compiled by Zung (1965), to measure depression. It comprises four subscales: psycho-emotional symptoms, physical diseases, psychomotor disorders, and psychological symptoms of depression. Higher scores indicate greater depression severity. We conducted a confirmatory factor analysis of the questionnaire using Amos software, and the results were as follows: Goodness of Fit Index (GFI) = 0.950, Incremental Fit Index (IFI) = 0.956, Comparative Fit Index (CFI) = 0.954, Tucker-Lewis index (TLI) = 0.927, and Root Mean Square Error of Approximation (RMSEA) = 0.039. In this study, the Cronbach’s α for the total scale was 0.768.

#### Social Support Rating Scale

We used the 10-item Social Support Rating Scale (SSRS), developed by Xiao (1994), to measure the degree of social support. It comprises three subscales: subjective support, objective support, and utilization of support. We conducted a confirmatory factor analysis of the questionnaire using Amos software, and the results were as follows: GFI = 0.973, IFI = 0.979, CFI = 0.978, Normed Fit Index (NFI) = 0.926, RMSEA = 0.033, and RMSEA = 0.039. In this study, the Cronbach’s α was 0.695.

#### Adult Attachment Scale

We used the English version of the 18-item Adult Attachment Scale (AAS), compiled by Collins and Read (1990), to measure attachment type. It comprises three subscales: attachment closeness, attachment dependence, and attachment anxiety. We conducted a confirmatory factor analysis of the questionnaire using AMOS software, and the results were as follows: GFI = 0.954, IFI = 0.943, CFI = 0.939, TLI = 0.901, and RMSEA = 0.043. In this study, the Cronbach’s α for the total scale was 0.628, and that for attachment closeness was 0.645.

#### Self-Esteem Scale

We used the English version of the 10-item Rosenberg Self-Esteem Scale (SES; Rosenberg, 1965) to measure self-esteem. It comprises two subscales: self-affirmation and self-denial. Higher scores represent higher self-esteem. We conducted a confirmatory factor analysis of the questionnaire using Amos software, and the results were as follows: GFI = 0.959, IFI = 0.935, CFI = 0.933, NFI = 0.911, and RMSEA = 0.085. In this study, the Cronbach’s α for this scale was 0.710.

### Statistical Analysis

We used SPSS 23.0 to perform descriptive statistics. Pearson correlation analysis was conducted, and Hayes SPSS macro program PROCESS was used to analyze the data. We also used Amos 22.0 to construct the structural equation model and test the bootstrap mediation effect. Due to the complexity of structural equation models, it is generally recommended to report detailed model fit indicators: absolute fit index GFI and RMSEA and relative fit index CFI, IFI, and CFI.

## Results

### Common Method Biases

We employed the Harman single factor method and conducted the common method bias test. The results showed that there were 15 factors with eigenvalues greater than 1 and the first factor explained 11.57% of the variance; this was less than the critical standard of 40%. Thus, our results suggested that there was no serious common method bias in our data.

### Correlation Analysis

Written informed consent was obtained before the experiments, and the study was approved by the committee of the ethnic board of Nanjing University of Chinese Medicine and the latest revision of the Declaration of Helsinki. With regard to the prevalence of depression, 99 (28.4%) out of the 349 students were mildly depressed, 94 (26.9%) were moderately depressed, 24 (6.9%) were severely depressed, and 132 (37.8%) were not depressed. According to past interviews, international students’ depressive symptoms tend to change at 6 months; as in Lysgaard’s U-curve theory, international students may face a cultural shock after a “honeymoon” period in a new country. This implies that international students residing in China for less than 6 months would have better psychological health than those residing for more than 6 months. Through this scheme, we tested the correlation between depression, social support, attachment closeness, and self-esteem (Table 1) and the mean and *SD* of all variables.

**TABLE 1:** Results of correlation analysis between all variables of interest.

Variables	Mean	*SD*	1	2	3	4
1. Depression	43.13	8.92	1			
2. Social support	33.99	6.20	−0.251**	1		
3. Attachment closeness	17.22	4.82	−0.198**	0.149**	1	
4. Self-esteem	29.98	5.35	−0.257**	0.116*	0.003	1

***P < 0.001 (two-tailed), **P < 0.01 (two-tailed), and *P < 0.05 (two-tailed).

There was a significant negative correlation between depression and social support, between depression and attachment closeness, and between depression and self-esteem, and there was a significant positive correlation between social support and attachment closeness and between social support and self-esteem. Attachment closeness is not related to depression.

### Construction of the Intermediary Model

**FIGURE 2:**
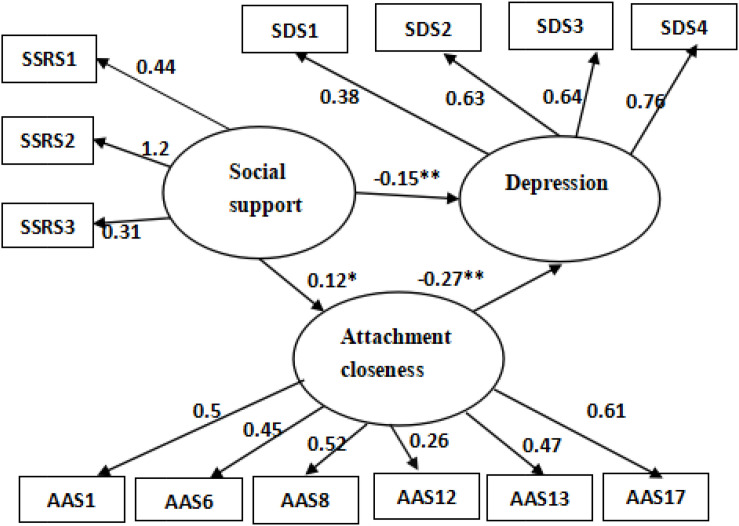
Path diagram of the relationship between depression, social support, attachment closeness, and length of study abroad. The figure shows all the standardized path coefficients in the model. *P < 0.05 (two-tailed) and **P < 0.01 (two-tailed).

The structural equation models of depression, social support, attachment closeness, and length of study abroad period are shown in Figure 2. The results yielded various fit indices: x^2^/df = 1.921, GFI = 0.946, Adjusted Goodness of Fit Index (AGFI) = 0.920, IFI = 0.923, TLI = 0.901, CFI = 0.921, and RMSEA = 0.051. Thus, the proposed structural equation model showed a good fit for all indices, indicating that the model was reasonable and could be used.

### Mediating Effect

The mediating effects of attachment closeness on the relationship between depression and social support are shown in Table 2. We used the bias-corrected non-parametric percentage bootstrap method to test this mediating effect. We calculated the 95% confidence interval (95% CI) and generated 5,000 repeat samples. The bias-corrected 95% CI for the direct effect was (−0.340, −0.049), and the percentile 95% CI was (−0.360, −0.059); namely, the results showed no zero value, indicating that there was a direct effect.

**TABLE 2:** The standardized estimates based on the bootstrap tests.

	Effect size	Bias corrected (95% CI)	Percentile (95% CI)
Total effect	−0.182	(−0.265, −0.058)	(−0.268, −0.059)
Direct effect	−0.150	(−0.340, −0.049)	(−0.360, −0.059)
Indirect effect	−0.032	(−0.086, −0.005)	(−0.080, −0.002)

The bias-corrected 95% CI for the indirect effect was (−0.086, −0.005), and the percentile 95% CI was (−0.080, −0.002); namely, the results showed no zero value, indicating that there was an indirect effect. Accordingly, attachment closeness played a partial mediating role in the relationship between social support and depression. The direct (−0.150) and indirect effects (−0.032) accounted for 82.42 and 17.58% of the total effects (−0.182), respectively.

### Moderating Effect

Regarding the moderating effect of self-esteem, the results showed that social support had a significant predictive effect on attachment closeness (β = 0.110, *P* < 0.001) and the interaction between social support and self-esteem had a significant effect on attachment closeness (β = 0.020, *P* < 0.05). The effect of social support on depression was significant (β = −0.325, *P* < 0.001), and the effect of attachment closeness on depression was also significant (β = −0.305, *P* < 0.05) (Table 3). Thus, we found that the moderated mediation model was supported, that is, the mediating effect of social support on depression was moderated by self-esteem.

**TABLE 3:** Results of the moderating mediation model test.

Equation 1 (effect standard: attachment closeness)				Equation 1 (effect standard: depression)			
	
Variable	β	*SE*	*t*	Variable	β	*SE*	*t*
Social support	0.110	0.041	2.667***	Social support	−0.325	0.075	−4.363***
Self-esteem	–0.003	0.048	–0.065	Attachment closeness	−0.305	0.096	−3.173**
Social support × self-esteem	0.020	0.007	2.947**				
R^2^	0.046				0.089		
F	5.591				16.986		

***P < 0.001 (two-tailed), **P < 0.01 (two-tailed), and *P < 0.05 (two-tailed).

**FIGURE 3:**
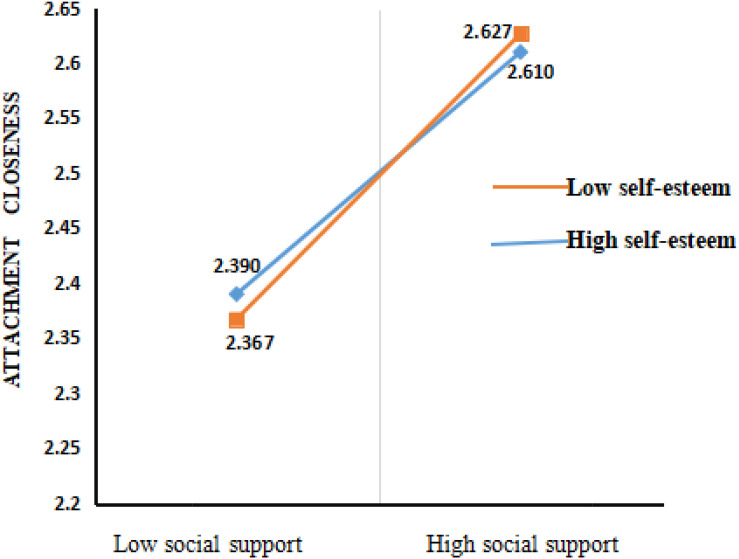
The moderating effect of self-esteem on the relationship between social support and attachment closeness.

To study the mediating effect value and 95% bootstrap confidence zone of attachment closeness under different self-esteem levels, we divided students based on their self-esteem scores into high, medium, and low self-esteem groups. To clarify the moderating effect of self-esteem, we conducted a simple slope test (Preacher et al., 2006). The results are illustrated in Figure 3. The results showed that, compared with students with low self-esteem, the predictive effect of social support on attachment closeness was enhanced in those with high self-esteem. We defined the cut-off points as follows: high self-esteem was defined as having a score higher than the average plus one standard deviation and low self-esteem was defined as having a score lower than the average minus one standard deviation. The 95% bootstrap CI of attachment closeness is shown in Table 4.

**TABLE 4:** The moderating effects of self-esteem on the mediation effect of attachment closeness.

Self-esteem level	Boot effect	Boot SE	Boot LLCI	Boot ULCI
M − *SD*	0.001	0.020	−0.046	0.035
M	–0.034	0.176	−0.791	–0.007
M + *SD*	–0.066	0.028	−0.136	–0.023

## Discussion

### The Relationship Between Depression and Social Support

Our results showed that there was a significant negative correlation between social support and depression among international students in China; that is, the more social support they had, the less likely they were to become depressed. Conversely, those with low social support were more likely to feel uncomfortable with the Chinese/new environment and to have more severe depressive symptoms. These findings were consistent with previous studies on the effect of college students’ social support on depression (Wang et al., 2014); for example, in one study, individuals with lower levels of social support were shown to be more likely to have depression (Reid et al., 2016). The authors of the current study have also conducted research on depression that produced results that concur with the current findings (Gu et al., 2019a,b; Zheng et al., 2019). Moreover, a recent report showed that there is a certain connection between depression and social support; specifically, individuals with less social support have more negative emotions (e.g., anxiety and depression). Together, these citations and our results underpin the fact that social support is closely related to depression, and that it plays a role in alleviating depression among international students. Since international students are far away from their homeland, family, and friends, their access to social support may be weakened, thereby making them more prone to depression.

### The Mediating Role of Attachment

This study also showed that attachment closeness not only directly affects depression but also directly intermediates the impact of social support on depression; this result may provide new directions for future studies on coping with depression, among international students in China. In other words, international students with low attachment closeness are more likely to have depression due to the lack of social support. When the relationship between attachment and closeness in reality is not satisfactory, they are more likely to reduce depression through social support. Specifically, foreign students who had been in an environment with high levels of social support for a long time were more willing to come into contact with others and to establish close relationships; this could gradually increase attachment closeness, allow them to have an easier way to get along with people around them, and make it easier for them to adapt to the Chinese environment. Together, these factors may help reduce the risk for depression.

Our results highlight that attachment closeness can relieve depression to a certain extent, and that social support might affect depression through attachment closeness. Therefore, when international students in China suffer from depression, stakeholders should pay attention to their attachment and social support levels; moreover, when developing interventions aimed at training students’ attachment closeness—which may improve students’ social support, thereby allowing for alleviation of their depressive symptoms—stakeholders should first apply comprehensive assessment methodologies to analyze students’ attachment issues.

**FIGURE 4:**
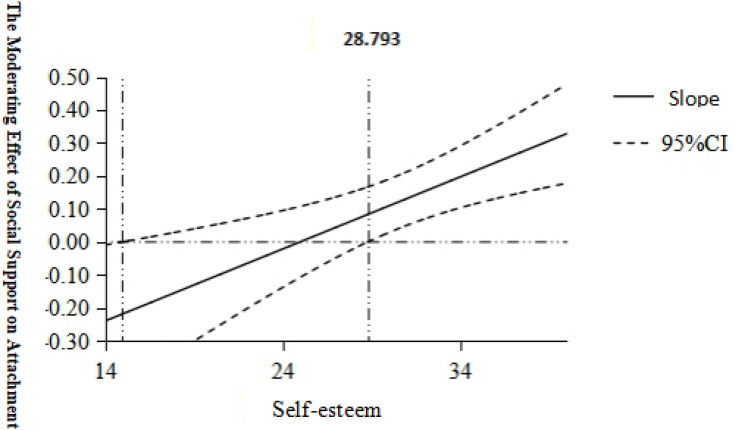
Visualized schematic diagram of the moderating effect of self-esteem on the effects of social support and attachment closeness.

To further explore the boundary value of the self-esteem moderation effect and the range of statistically different self-esteem values, we used the PROCESS program to carry out the Johnson–Neyman technique test; the results are shown in Figure 4. We found that when the value of self-esteem was greater than 28.793 in the 95% CI, the moderating effect was significant. Specifically, when the total score of self-esteem was less than this value, the moderating effect of self-esteem was significant.

To conclude, our results denote that low social support can directly predict depression, and can also affect depression through the mediating effect of attachment closeness, in international students in China. Demonstrating this mediation model is significant because it shows that depression in this population is not only affected by internal (i.e., attachment closeness) but also affected by external factors (i.e., social support). Reflecting the ecological psychology theory, human behavior is the result of the interaction between individual internal and external factors.

### The Moderating Effect of Self-Esteem

Previous studies have examined the relationship between self-esteem and depression (Sowislo and Orth, 2013; Li et al., 2019; Shen et al., 2019). Most studies on adult attachment are based on two dimensions of this construct, namely, attachment anxiety and avoidance, whereas there are few studies exploring the attachment closeness dimension (Valikhani et al., 2018). First, our results demonstrated that the direct effect of social support on depression and the mediating effect of attachment are regulated by self-esteem; then, further analyses showed that although self-esteem had a moderating effect on the first half of the pathway, it had an insignificant moderating effect on the second half (i.e., the interaction between self-esteem and social support). Moreover, our analyses showed that although moderate and high levels of self-esteem played an indirect role in the effect of social support on depression, low self-esteem did not affect the mediated relationship we analyzed.

Therefore, compared with individuals with low self-esteem, the mediating effect of attachment and closeness is stronger in international students with high self-esteem, which means that high self-esteem enhances the impact of depression. International students with high self-esteem are better able to cope with depression and take the initiative to adjust and relieve the problems caused by depressive symptoms, whereas international students with low self-esteem are more susceptible to depression and are, therefore, more likely to rely on the mediating role of attachment closeness to relieve depression. International students in China may be more prone to depression owing to having to deal concomitantly with cultural shock, new customs, and academic pressure. International students with high self-esteem can better cope with depressive emotions, and attachment closeness can have a certain buffer effect on the depressive symptoms of these students.

## Conclusion

In this study, we proposed that attachment closeness would be a mediator in the relationship between social support and depression, and that this mediating effect would be moderated by self-esteem. In our results, the mediating effect was stronger in individuals with high self-esteem than in those with low self-esteem; therefore, this hypothesis was confirmed. To the best of our knowledge, this study is the first to demonstrate the moderating role of self-esteem for the mediation effect of attachment closeness in the relationship between social support and depression. Additionally, we found that if international students are exposed to environments in which they receive social support and feel comfortable, they may be more willing to establish good relationships with others and become attached to them; accordingly, such attachment may reduce depressive symptoms evoked by being away from their home and families, which may ultimately promote their physical and mental health. However, this study also has certain limitations. We did not inquire whether the students were in a relationship or were married; we did not investigate the cultural background of the foreign students in their home countries or their previous study abroad experience. Future studies should incorporate these variables in studying the relationship between depression and social support among international students in China.

## Data Availability Statement

The original contributions presented in the study are included in the article/supplementary material, further inquiries can be directed to the corresponding author/s.

## Author Contributions

YawL and SG designed the study. YawL, FL, and QX conducted the survey. YW, YanL, and ZZ analyzed the data. YawL, SG, and ZZ wrote the manuscript. All authors contributed to the article and approved the submitted version.

## Conflict of Interest

The authors declare that the research was conducted in the absence of any commercial or financial relationships that could be construed as a potential conflict of interest.
